# Inhibitory Potential of* Acroptilon repens* against Key Enzymes involved in Alzheimer and Diabetes, Phytochemical Profile, Radical Scavenging, and Antibacterial Activity 

**DOI:** 10.29252/ibj.25.1.21

**Published:** 2020-08-25

**Authors:** Moslem Afsharnezhad, S. Shirin Shahangian, Behnam Rasti, Mohammad Faezi Ghasemi

**Affiliations:** 1 Department of Biology, Faculty of Sciences, University of Guilan, Rasht, Iran;; 2 Department of Microbiology, Faculty of Basic Sciences, Lahijan Branch, Islamic Azad University (IAU), Lahijan, Guilan, Iran

**Keywords:** Acroptilon repens, Antioxidants, Phytochemicals

## Abstract

**Background:**

This study was devoted to assessing the inhibitory potential of acetone, methanol, and ethanol extracts of *Acroptilon repens* against disease-associated enzymes, as well as their antioxidant/antibacterial activity and phytochemical composition.

**Methods:**

Comparative assessment using various antioxidant evaluation methods, including FRAP, scavenging ability on DPPH radical and hydrogen peroxide, and RP, indicated that the acetone extract presented the highest antioxidant activity, due to its highest total antioxidant content.

**Results:**

The TPC and TFC of these extracts were 3.44 ± 0.32 mg GAE/g DW and 2.09 ± 0.2 mg QE/g DW, respectively. The hydrodistillation essential oil from *A. repens* was analyzed by GC/MS, and 17 compounds were identified. All extracts showed good inhibitory activities against disease-related enzyme acetylcholinesterase and α-amylase, with the lowest IC_50_ for acetonic extract. Extracts of *A. repens* exhibited inhibiting activities against the Gram-positive bacteria, with the most effect of acetone extract.

**Conclusion:**

Our findings suggest *A. repens* as a promising source of natural antioxidant, antimicrobial, anti-cholinesterase and anti-amylase agents for the management of oxidative damage, and pharmaceutical, food, and cosmeceutical purposes.

## INTRODUCTION

Due to the valuable roles of plant secondary metabolites in preventing chronic disorders, the number of research activities on medicinal and aromatic plants have highly increased in recent years^[1]^. As a well-known type of secondary metabolites, polyphenolic compounds possess several biological effects, including antioxidant, antibacterial, antimutagenic, and anti-inflammatory activities^[^^2^^]^. 

Oxidative stress is resulted from a shift in the balance between the antioxidant defense systems and the formation of ROS, in favor of the latter. ROS has critical roles in the occurrence of various diseases, including cancer, atherosclerosis, neurodegenerative disorders (Alzheimer's and Parkinson's diseases), and diabetes mellitus^[^^3^^]^. Phenolic compounds act as the scavengers of these harmful free radicals, thereby preventing or reversing their adverse effects^[^^4^^]^. Synthetic antioxidants such as, tertiary butyl hydroquinone, BHA, and butylated hydroxyltoluene are widely used in food industry^[^^5^^]^. Long-term consumption of synthetic antioxidants, however, is always accompanied with various risks for health^[^^6^^]^. To partly overcome the side effects of synthetic antioxidant compounds, considerable attention has been paid to the use of natural ingredients. Many researchers have recently focused on plant-derived metabolites. Besides the antioxidant activity, the secondary metabolites could also play other different roles, including inhibition of certain disease-associated enzymes. For instance, voglibose, galantamine, and kojic acid are currently used as pharmaceuticals to reduce the actions of α-amylase, cholinesterase, and tyrosinase, respectively^[^^7^^]^. Nonetheless, due to their side effects, many attempts have been made to find more efficient and safe alternatives, notably from natural sources^[^^8^^]^. 

The type of solvent employed for the extraction could affect the antioxidant activity of extracts. Several extracting solvents or their mixture are generally applied with trial and error to finally discover the best conditions for obtaining specific compounds^[^^9^^]^. The aqueous mixtures of ethanol, methanol, and acetone are commonly used as the most appropriate solvents^[^^10^^]^.

*Acroptilon repens* (L.) DC belongs to the family of Asteraceae and is a perennial herbaceous plant. It is found in many areas in the world, including Iran, Turkish Armenia, Western Turkestan and, Mongolia^[^^11^^]^. In folk medicine, the *A. repens* is used for many years as an emetic, anti-epileptic and anti-malaria remedy. It has been shown that the essential oil could control the activity of the Epstein-Barr virus due to the presence of a triterpene called euphorbol^[^^12^^]^. Only limited information about *A. repens *has previously been reported^[^^13^^,^^14^^]^. The main objectives of the present research were (i) to identify the chemical compositions of the essential oil of *A. repens* by GC-MS, (ii) to determine the effect of different extraction solvents on the bioactive components (TPC, TFC, and TAC) and antioxidant activity of *A. repens*, (iii) to evaluate the enzyme inhibitory and (iv) antibacterial activities of various extracts of *A. repens*. 

## MATERIALS AND METHODS

**Chemicals**

Folin-Ciocalteu reagent, DPPH, DTNB, DNS, TPTZ, gallic acid, quercetin, pancreatic α-amylase (3.2.1.1), and AChE from *Electrophorus electricus*‎ (3.1.1.7) were purchased from Sigma Aldrich (St. Louis, MO, USA). FeCl_3_, AlCl_3_.6H_2_O, K_3_Fe(CN)_6_, and ammonium iron (II) sulfate hexahydrate, (NH_4_)_2_Fe(SO_4_)_2_.6H_2_O, were obtained from Merck (Darmstadt, Germany). All other chemicals and solvents (such as methanol, acetone, and ethanol) were of analytical grade purity. The specific buffers were freshly prepared in our biochemistry laboratory.

**Sample preparation**

The leaves of *A. repens* were collected from Neyriz, east of Fars Province (Iran), in April 2019. The air-dried and grounded leaves of *A. repens* were extracted using 70% aqueous solution of ethanol, methanol, and acetone. Then 10 g of dry powder of leaves was separately subjected to solvent extraction using 100 ml of solvents for one day. The mixture was then filtered on Whatman No. 1 filter paper, and a second extraction was performed. Both supernatants were mixed and concentrated in vacuum at 40 °C. The extraction for each solvent was conducted three times, and the obtained extracts were stored at 4 °C until further analysis.

**Preparation of essential oil**

The essential oil was obtained from 250 g of leaves of *A. repens* plant by hydrodistillation method using a clevenger type apparatus for 3.5 h. The oil was dried over anhydrous sodium sulfate and stored 4 °C until analysis.

**GC-MS analysis**

The chemical compositions of the essential oil were determined by GC-MS on a HP-6890/HP5 equipment (Agilent-7890A, Madrid, Spain). The GC was equipped with a TRB-5MS column (32 m × 0.25 mm × 0.25 mm). The carrier gas was helium, wirh the split injection of 1 ml at 1 ml/min flow rate, and the injection temperature of 60 - 220 °C, with an increasing rate of 6 °C/min. The chemical constituents of the oil were identified by comparing their mass spectra with those available in the Wiley GC-MS library as well as by comparing them with those reported in the literature^[^^13^^,^^14^^]^.

**Determination of TPC**

A former method was applied with some modifications to estimate the TPC of extracts^[^^15^^]^. Briefly, 100 µl of standard solutions (gallic acid) or appropriately diluted extracts were mixed with 100 µl of Folin-Ciocalteu reagent, 2 ml of 2% sodium carbonate, and 2.8 ml of deionized water. Following the incubation of mixtures at room temperature for 30 min, the absorbance was recorded at 720 nm by a UV-Vis spectrophotometer. TPC was expressed as mg of gallic acid equivalent per g DW (mg GAE/g DW).

**Determination of TFC**

The aluminum chloride colorimetric assay was used as explained before to determine TFC with minor changes^[^^16^^]^. Quercetin was employed as a standard. The diluted extracts or quercetin (0.5 ml) were mixed with 2.8 ml of distilled water, followed by the addition of 100 µl of 10% (w/v) aluminum chloride and 100 µl of potassium acetate (1.0 M) solution. After 40 min of incubation, the absorbance was read at 415 nm. The standard curve of quercetin was utilized to determine the TFC expressed as mg quercetin equivalent/gDW.

**Determination of** **TAC**

TAC was determined by a previously described method^[^^17^^]^. Leaves of *A. repens* were homogenized in acidified solvents (ethanol, methanol, and acetone containing 1% HCl [v/v]) and kept at 4 ºC for 24 h. The extracts were later centrifuged, and the absorbance of supernatants was read at 530 and 675 nm. TAC of all the extracts was calculated according to Eq. (1).

Anthocyanin content (mg/gDW) = A_530_ - (0.25 × A_675_) (1)

Where A_530 _and A_675_ are the extract absorption in 530 nm and 657 nm, respectively.

**DPPH free radical scavenging activity**

The scavenging ability of extracts against DPPH radical was evaluated by an earlier method with some changes^[^^18^^]^. In brief, 1 ml of various concentrations (0.25-4 mg/ml) of each extract was mixed with 1 ml of 0.1 mM DPPH solution in methanol. The mixture was allowed to stand (0.5 h/in the dark), and the absorbance was measured at 517 nm against the blank (methanol). Then 0.1 mM of DPPH solution was used separately as the control. The inhibition percentage for scavenging DPPH radical by the extracts was calculated according to Eq. (2).

 % inhibition= (A_control_-A_sample_)/A_control_ ×100 (2)

Where A_control _and A_sample_ are the absorbances of the DPPH solution in the absence and presence of extract, respectively. The results were plotted against the concentration of each sample to calculate the IC_50_ value. BHA was used as the positive control.

**FRAP assay**

FRAP assay was carried out the same as before with some changes^[^^19^^]^. Briefly, FRAP reagent was prepared by mixing acetate buffer (300 mM, pH 3.6), TPTZ (10 mM in 40 mM HCl), and FeCl_3_ (20 mM) in a ratio of 10:1:1. Afterwards, 500 µl of each sample was mixed with 1.5 ml of FRAP reagent. The mixture was shaken vigorously and incubated in the dark at room temperature for 30 min before measuring the absorbance at 593 nm. Ferrous ammonium sulfate hexahydrate was used to create the standard curve. The FRAP value was expressed as mM FeSO_4_ equivalents per gram of DW.

**H**_2_ **O**_2_ **assay**

The ability of different extracts for H_2_O_2_ scavenging was evaluated as described before^[^^20^^]^. Briefly, all extracts (1.5 ml) were mixed with 20 µl of H_2_O_2_ solution 30%. The absorbance was monitored at 530 nm at 5-min intervals until it remained constant. The decrease in the absorbance was proportional to the H_2_O_2_ scavenging ability of extracts. The percent inhibition of H_2_O_2_ by the extracts was calculated according to Eq. (3).

%Inhibition of H_2_O_2_= (A_control_-A_sample_)/A_control_ ×100 (3)

Where A_control _and A_sample_ are the absorbances of the extracts in the absence and presence of H_2_O_2_, respectively. Finally, IT_50_ was determined as the time needed to dispose 50% of H_2_O_2_ radicals.

**RP assay**

A formerly described protocol was used to evaluate the ferric RP of the extracts^[^^21^^]^. For this purpose, 1 ml of various concentrations (0.1-1 mg/ml) of each extract was mixed with 1 ml of phosphate buffer (0.2 M, pH 6.6) and 1 ml of potassium ferricyanide (1%), followed by incubating at 50 °C for 20 min. Then 1 ml of trichloroacetic acid (10%) was added to the mixture and centrifuged (3000 ×g, 10 min, 4 °C). After mixing 1.5 ml of supernatant with distilled water (1.5 ml) and FeCl_3_ (0.15 ml, 0.1%), the mixture was incubated at room temperature for 10 min, and the absorbance was read at 700 nm. The higher the absorbance of the reaction mixture, the higher the RP of the extracts. The extract concentration providing EC_50_ was calculated from the graph of absorbance at 700 nm against the extract concentration. Trolox under the same conditions was used as the positive control.

**Enzyme inhibitory activity**

***AAI***

The inhibitory effect of the extracts on α-amylase activity was measured as stated earlier^[^^22^^]^ with some modifications. To do this, 50 μl of different concentrations (0.048 to 0.8 mg/ml) of extracts and enzyme solutions were mixed and pre-incubated at 37 °C for 15 min. Afterward, the reaction was *initiated* by adding 100 μl of starch solution. After the incubation of mixture at 37 °C for 3 min, the reaction was stopped by adding 200 μl of DNS and boiled for 5 min. Next, the mixture was diluted with distilled water (3.6 ml), and the absorbance was recorded at *540 nm* against the blank. The control sample was prepared with the same procedure, adding distilled water instead of the extracts. The α-amylase inhibitory activity of the extracts was calculated in terms of the inhibition percentage using Eq .(4).

 %I_α-amylase_ = (A_control_-A_sample_)/A_control_ ×100 (4)

The %I_α-amylase _was plotted against the concentration of the extracts to calculate the IC_50_ value. Acarbose was used as the positive control.

***AChE inhibition***

The inhibition effects of various extracts on AChE activity was determined as per a modified method^[^^23^^]^. At first, 200 µl of DTNB (5 mM), 150 µl of Tris/HCl buffer (50 mM, pH 7.4), 10 µl of AChE, and 50 µl of different concentrations (0.1-1.5 mg/ml) of extracts were mixed and incubated at room temperature for 10 min. After incubation, the enzyme reaction was initiated by adding 100 µl of acetylthiocholine chloride. Control was prepared using the same procedure without any plant extracts. The hydrolysis of the substrate was recorded at 410 nm. AChE inhibitory activity was calculated according to Eq. (5) 

 %I_AChE_ = (A_control_-A_sample_)/A_control_ ×100 (5)

Based on the obtained results, the value of IC_50_ was calculated by plotting the concentrations of the extracts versus %I_AChE_. Galanthamine was used as the positive control.

**Determination of antibacterial activity**

The antimicrobial activity of three extracts of *A. repens* against Gram-negative (*Pseudomonas aeruginosa* PTCC-1620 and *Escherichia coli* PTCC-1338) and Gram-positive bacteria (*Staphylococcus aureus* PTCC-1430, *Micrococcus luteus* PTCC 1625, and *Bacillus* *subtilis* PTCC-1720) was evaluated using the agar based-disk diffusion method. Inoculum density of the bacteria was prepared equivalent to 0.5 McFarland standard and uniformly spread on the Luria-Bertani agar plates. The sterile discs (6.0 mm) were placed over plates, and 20 μl of the test sample was placed over the discs. After that, the plates were kept in a refrigerator at 4 °C for 60 min, followed by further incubation at 37 °C for 24 h. Evaluation of antibacterial activity was performed by measuring the zones of growth inhibition around the disks.

**Statistical analysis**

All the data were reported as mean ± SD (standard deviation) and carried out with three replicates. Statistical analyses were performed with ANOVA and Tukey’s post hoc test with *p* < 0.05. Besides, the two-tailed Pearson’s correlation test determined later the existence of the possible relationships between parameters. All the analyses were carried out by using SPSS version 23.0 software (SPSS Inc., Chicago, IL).

## RESULTS

**Total bioactive compounds**

As the first step of our experimental process, amounts of total bioactive compounds were evaluated in various extracts of *A. repens* (Table 1). The results showed that the TPC in various extracts presented significant differences according to the solvent used. The highest content of TPC was obtained by 70% acetone, followed by the 70% methanol. The extract prepared with 70% ethanol presented the lowest content. The effect of the solvents on TFC of extracts followed a similar trend to total phenol, as the 70% acetonic extract has the highest level of flavonoid content, followed by the 70% methanol and 70% ethanol extracts. The most efficient solvent for extracting anthocyanins was the acidified methanol. 

**Antioxidant activity**

**Fig. 1:**
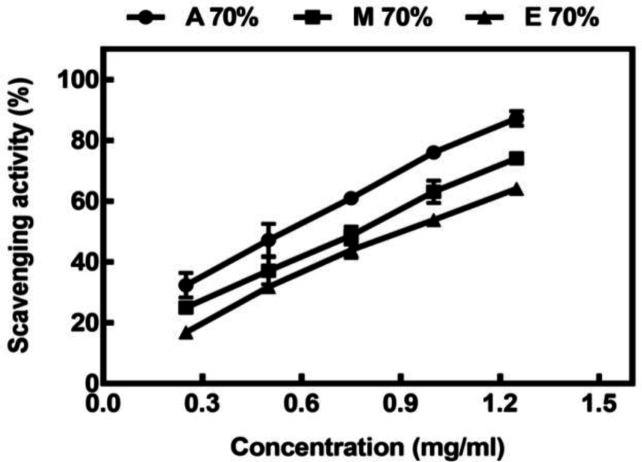
DPPH radical scavenging activity of different extracts of *Acroptilon repens*. A 70% (acetone 70%), M 70% (methanol 70%), and E 70% (ethanol 70%).

**Fig. 2:**
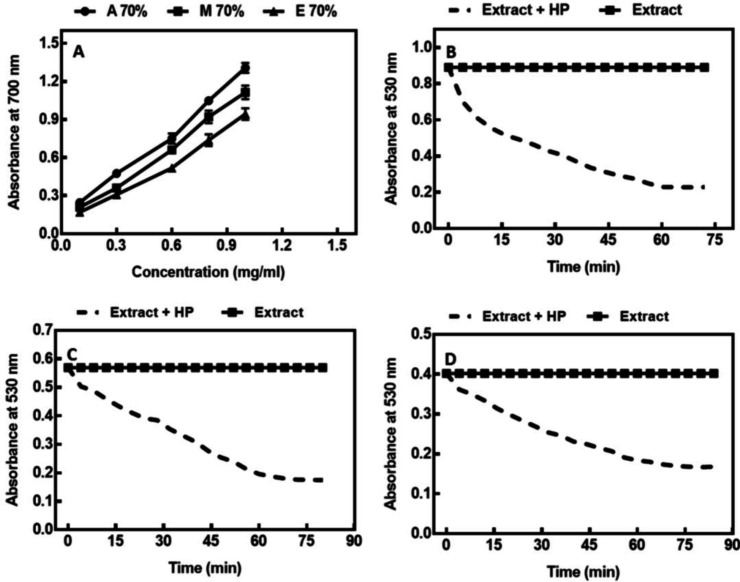
(A). RP of different extracts (A 70%, acetone 70%; M 70%, methanol 70%; E 70%, ethanol 70%). Hydrogen peroxide scavenging ability of different extracts: (B) acetone, (C) methanol, and (D) ethanol. HP, hydrogen peroxide

The antioxidant activity of *A. repens* was assessed by evaluating H_2_O_2_ and DPPH radical scavenging ability and RP assays (FRAP and RP), and the results are represented in Table 2. As revealed in Figure 1, all the extracts showed dose-response scavenging activity on DPPH. The ability of plant extracts to scavenge the DPPH radical is presented by the IC_50_ value. The lower is the IC_50_ value, the stronger is the antioxidant activity of extracts. It was found that the type of solvent significantly affects the DPPH scavenging activity of *A. repens*. The extracts obtained using 70% acetone exhibited the highest DPPH scavenging capacity (IC_50_: 0.551 ± 0.1 mg/ml), followed by the 70% methanol extract. The weakest DPPH scavenging capacity was obtained for the 70% ethanol extracts. However, the synthetic antioxidant, BHA, exhibited a higher DPPH radical scavenging effect than all *A. repens* extracts. The FRAP values of different extracts were consistent with the antioxidant potential obtained from the DPPH assay. The result emphasized that 70% acetone extract possessed the strongest ferric RP as compared to other extracts (0.74 ± 0.049 mM Fe(II)/gDW). The weakest capacity to ferric RP was obtained in the ethanolic extract. The RP assay was peformed based on the redox reaction of the ferric ion in the presence of an antioxidant. The presence of antioxidants in the extracts led to the conversion of the oxidized Fe^3+^/ferricyanide complex to its reduced form, where the formation of Perl’s Prussian Blue was monitored at 700 nm. The higher absorbance of the reaction mixture indicated its higher reducing ability. As depicted in Fig. 2A, three extracts of *A. repens* showed a concentration-dependent increase in their RP. The extract obtained with acetone exhibited the higher RP compared to those obtained with two other solvents. The EC_50_ values of RP are presented in Table 2. The hydrogen peroxide scavenging ability of different extracts of *A. repens* is indicated in Fig. 2A, 2B, and 2C. An apparent decrease in absorbance at 530 nm in the presence of H_2_O_2_ reflected the scavenging ability of the extracts. During this process, the phenolic compounds present in the extracts were oxidized, resulting in the discoloration as mentioned earlier. IT_50_ (the time needed to dispose 50% of H_2_O_2_ radical) is another parameter for the evaluation of the inhibitory effect of H_2_O_2_ radical, where the lower the IT_50_ value, the stronger the antioxidant activity. As shown in Table 2, the extracts obtained using 70% acetone exhibited the highest hydrogen peroxide scavenging ability, followed by the 70% methanol extract and 70% ethanol. A previous study regarding the effect of solvents on the extraction of phenolic compounds of the eggplant byproduct showed a similar trend for hydrogen peroxide scavenging ability^[^^20^^]^.

**Table 1:** TPC, TFC, and TAC of *Acroptilon repens*, extracted using various solvents

**Solvent (70%)**	**TPC** **(mg GAE/g DW)**	**TFC** **(mg QE/g DW)**	**TAC** **(mg/g DW)**
Acetone	3.44 ± 0.32^a^	2.09 ± 0.2^a^	0.26 ± 0.02^c^
Methanol	2.45 ± 0.27^b^	1.43 ± 0.15^b^	0.59 ± 0.035^a^
Ethanol	2.32 ± 0.31^b^	1.02 ± 0.08^c^	0.47 ± 0.03^b^

Each value is expressed as mean ± SD (n = 3). Means, in the same column, with different letters are significantly different (*p* < 0.05).

**Table 2:** Effect of the extraction solvent on the antioxidant activities of *Acroptilon repens*

**Solvent**	**DPPH** **(IC**_50_ **, mg/ml)**	**FRAP ** **(mM Fe (II)/g DW)**	**RP** **(EC**_50_ ** mg/ml)**	**Scavenging effect of H**_2_ **O**_2_ **IT**_50_** (min)**
Acetone (70%)	0.551 ± 0.1^a^	0.74 ± 0.049^a^	0.354 ± 0.01^a^	30.06 ± 1.73^a^
Methanol (70%)	0.756 ± 0.12^b^	0.59 ± 0.033^b^	0.425 ± 0.015^b^	43.57 ± 2.23^b^
Ethanol (70%)	0.918 ± 0.15^c^	0.45 ± 0.044^c^	0.528 ± 0.018^c^	51.18 ± 2.87^c^
BHA	0.03 ± 0.001	-	-	-
Trolox	-	-	0.012 ± 0.001	-

Each value is expressed as mean ± SD (n = 3). Means, in the same column, with different letters are significantly different (*p *< 0.05).

**Chemical composition of plant essential oil**

The essential oil from medicinal plants is complex mixtures of volatile secondary metabolites. In this study, analyzing the essential oil obtained by hydrodistillation of the leaves of *A. repens* by GC-MS led to the identification of 17 compounds. As shown in Table 3, the major compounds of essential oil were β-Cubebene (3.8%), ∆-Cadinene (4.1%), Germacrene D (4.4%), β-Caryophyllene (8.2%), α-Copaene (10.1%), and Caryophyllene oxide (12.7%).

**Enzyme inhibitory activity**

**Fig. 3:**
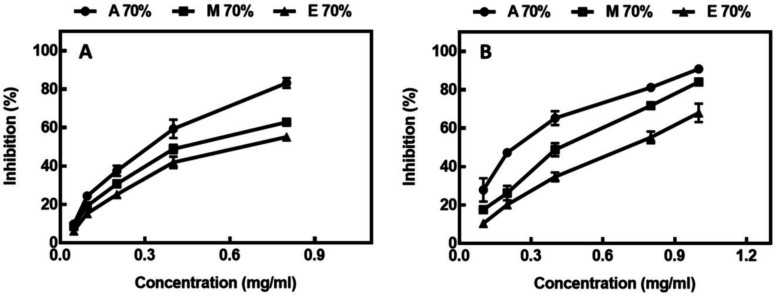
The effect of various extracts of *Acroptilon repens* on (A) α-amylase and (B) AChE activity. A 70%, acetone 70%; M 70%, methanol 70%; E 70%, ethanol 70%

In this study, an *in vitro* inhibitory effect of different extracts of *A. repens* on α-amylase and AChE was investigated. As revealed in Figures 3, all the extracts showed concentration-dependent inhibitory activity. The ability of different extracts to inhibit α-amylase and AChE is presented by the IC_50_ value. As illustrated in Table 4, the AAI rate of the acetone extract was significantly higher than the methanol and ethanol extracts. On the other hand, all the *A. repens* extracts possessed promising inhibitory activity on AChE; among them, acetone suggested the best inhibitory effect, followed by methanol and ethanol extracts.

**Antibacterial activity**

The evaluation of the antibacterial effect of all three extracts of *A. repens* was screened against some Gram-negative and Gram-positive bacteria, which are well-known foodborne pathogens. All the extracts *significantly inhibited *the growth of Gram-positive bacteria in a dose-dependent manner (Table 5). However, no effect was observed against Gram-negative bacteria. The acetone extract showed its highest inhibitory effects against *B. subtilis* with the inhibition zone diameter of 25.0 mm, followed by *S. aureus* and *M. luteus* (inhibition zones of 22.0 and 18.0 mm, respectively). Following a similar trend, the methanol extracts exerted higher inhibitory activity against *B. subtilis* than *S. aureus* and *M. luteus* with the inhibition zones of 22.0, 19.0, and 16.0 mm, respectively. 

## DISCUSSION

A number of studies have been carried out on the chemical constituents of essential oil of *A. repens* from other regions^[^^24^^,^^25^^]^. In spite of some similarities, the samples obtained from different locations had substantial qualitative and quantitative differences. Caryophyllene oxide, as an oxygenated terpenoid, exhibited diverse biological (antimicrobial, antifungal, and anti-inflammatory) activities^[^^26^^,^^27^^]^. It has also been reported that caryophyllene oxide inhibits cell growth in human prostate and breast cancer cells by inducing apoptosis and suppressing the PI3K/AKT/ mTOR/S6K1 pathways^[^^28^^]^. In addition, Germacrene D and β-Caryophyllene, as other major components of the *A. repens*, possess potent, antimicrobial activity^[^^29^^]^. Dahham *et al.*^[^^30^^]^ showed that β-caryophyllene has selective antibacterial activity and more pronounced antifungal activity.

**Table 3:** Chemical composition of the essential oil of *Acroptilon repens*

**Compound**	**RI**	**Percentage**
Decanal	1198	0.5
α-Cubebene	1351	0.3
Cyclosativene	1367	0.1
α-Copaene	1378	10.1
β-Cubebene	1391	3.8
α-Gurjunene	1408	0.3
α-Cedrene	1411	2.7
β-Caryophyllene	1421	8.2
α-Humulene	1453	1.8
γ-Muurolene	1479	1.3
Germacrene D	1486	4.4
Cadina-1,4-diene	1503	0.8
γ- Cadinene	1516	1.7
∆-Cadinene	1521	4.1
δ- Cadinene	1526	0.9
Caryophyllene oxide	1578	12.7
1- Heptadecene	1691	3.1
Aliphatic hydrocarbons		7.0
Oxygenated sesquiterpenes		17.3

RI, retention indices

Phenolic compounds are one of the most important natural antioxidants because of their important bioactivities. Significant differences have been observed between TPC of various extracts of *A. repens*, in a way that the acetonic extract exhibited a meaningful difference compared to two other extracts (*p* < 0.05). No significant difference was observed between methanolic and ethanolic extracts. It seems that acetone is the most appropriate solvent for the extraction of phenolic compounds. In support of our results, other studies showed that acetone and ethanol are the most and least effective solvents for extracting phenolic contents from different plant sources^[^^20^^,^^31^^]^. Accordingly, acetone solvents could be the best choice when the extraction of phenolic contents is the main aim.

Due to the free radical scavenging ability, flavonoids are considered as antioxidants that can inhibit lipid oxidation in cell membranes or chelate metal ions^[^^32^^]^. Our results clearly demonstrated that the TFC was dependent on extraction solvents, introducing acetone as the best extracting solvent. Our results are similar to those of Sulaiman *et al.*^[^^33^^]^, who indicated that in different species of vegetable, the highest TFC was obtained mainly from the acetone extract. Also, Zieliński and Kozłowska^[^^34^^]^ showed that 80% acetone is the most efficient solvent for extracting flavonoids from cereal seeds.

 The beneficial health (antioxidant and anti-inflammatory) or nutraceutical effects of anthocyanins on visual capacity, brain cognitive function, obesity, cardiovascular risk, and cancer prevention notified the importance of the efficient extraction of these compounds. Accordingly, three solvents (70% acidified methanol, 70% acidified ethanol, and 70% acidified acetone) were chosen here. Acidified organic solvents, especially methanol, are widely used for the extraction of anthocyanins from plant materials^[^^35^^]^. The acidic nature of methanol can dissolve and stabilize the anthocyanins after the disruption of cell membrane^[^^36^^]^.

**Table 4:** Enzyme inhibitory activity of different extracts of *Acroptilon repens*

**Assays**	**IC**_50_ ** (mg/ml)**				
	**Acetone**	**Methanol**	**Ethanol**	**Galanthamine**	**Acarbose**
α-amylase	0.399 ± 0.016^a^	0.579 ± 0.02^b^	0.63 ± 0.028^c^	**-**	0.023 ± 0.005
Acetyl cholinesterase	0.325 ± 0.026^a^	0.448 ± 0.012^b^	0.621 ± 0.022^c^	0.018 ± 0.004	**-**

Each value is expressed as mean ± SD (n = 3). Means, in the same row, with different letters are significantly different (*p* < 0.05).

**Table 5:** Anti-bacterial activity of various extracts of *Acroptilon repens* at different concentrations against Gram-positive and Gram-negative bacterial strains

**St.**	**Acetone (mg/ml)**				**Methanol (mg/ml)**				**Ethanol (mg/ml)**		
	**150**	**100**	**50**		**150**	**100**	**50**		**150**	**100**	**50**
*BS*	25.0 ± 1.4^a^	19.0 ± 1.4^b^	15.0 ± 0.7^c^		22.0 ± 1.4^a^	17.0 ± 0.7^b^	14.0 ± 0.7^c^		18.0 ± 0.7^a^	14.0 ± 0.3^b^	12.0 ± 0.3^c^
*SA*	22.0 ± 0.7^a^	17.0 ± 0.3^b^	14.0 ± 0.3^c^		19.0 ± 1.4^a^	13.0 ± 0.7^b^	10.0 ± 0.3^c^		16.0 ± 0.7^a^	11.0 ± 1.4^b^	8.0 ± 1.4^c^
*ML*	19.0 ± 0.7^a^	16.0 ± 0.3^b^	13.0 ± .03^c^		16.0 ± 0.7^a^	12.0 ± 0.3^b^	9.0 ± 0.3^c^		14.0 ± 1.4^a^	10.0 ± 0.7^b^	7.0 ± 0.7^c^
*EC*	0	0	0		0	0	0		0	0	0
*PA*	0	0	0		0	0	0		0	0	0

Each value is expressed as mean ± SD (n = 3). Different letters in different extracts within the same row indicate significant differences (*p* < 0.05). St., strains; BS, *B. subtilis*; SA, *S. aureus*; ML, *M. luteus*; EC, *E. coli*; P*.a*., *P. aeruginosa*

Our findings were consistent with the results of some previous studies^[^^19^^,^^20^^]^. The antioxidative potential of plant extracts can be measured using various *in vitro* assays, and each assay is based on at least one feature of antioxidant activity. The DPPH method is widely used to evaluate the antioxidant capacity of plant extracts. The maximum absorption of DPPH at 517 nm decreases when the stable radical is reduced to the DPPH by accepting an electron or hydrogen atom from an antioxidant^[^^37^^]^. Based on the results, the acetone extract of *A. repens* exhibited the best DPPH scavenging capacity. It is worth mentioning that the type of extracted antioxidants depends on polarity, viscosity, and vapor pressure of the solvents. It has been demonstrated that the fewer viscose solvents can diffuse more easily into the plant pores, leading to the extraction of more diverse bioactive secondary metabolites^[^^36^^]^. In accordance with a number of studies, aqueous acetone is the most appropriate solvent for the high efficient extraction of antioxidant compounds and subsequent antioxidant activity. Zhao *et al.*^[^^38^^]^ have reported that the acetone/water solvent system is highly preferred to acquire the best antioxidant activity of *Hordeum vulgare* L. According to Kchaou *et al.*^[^^39^^]^, the extracts obtained using aqueous acetone solvent displayed higher DPPH radical scavenging activity compared to other solvents, which is in agreement with our results.

The electron-donating ability of antioxidants is widely evaluated by FRAP assay. In the presence of antioxidants, Fe^3+^-TPTZ complex reduces to Fe^2+^-TPTZ, creating a blue color with strong absorption at 593 nm^[^^40^^]^. The order of the antioxidant activity was: 70% acetone> 70% methanol > 70% ethanol (*p* < 0.05). The difference is probably due to the degree of solubility of phenolic compounds in different solvents. A similar trend has been reported in the study of the ferric reducing activity of *Etlingera elatior* and pineapple crude extract, supporting our data ^[^^19^^,^^41^^]^.

The RP assay of the extract, which may serve as a reflection of its antioxidant activity, was determined using a Fe^3+^ to Fe^2+^ reduction assay, whereby the yellow color of the test solution changes to various shades of green and blue, depending on the RP of the sample. The RP of three extracts was significantly (*p* < 0.05) different, according to the solvent used for extraction. The highest amounts of phenols and flavonoids were found in the aqueous acetone extract of *A. repens* (Table 1), which resulted in the higher RP of this extract in comparison with methanol and ethanol extracts. As per this significant correlation, phenolic compounds may contribute to the RP of extracts. However, their chemical structure could influence the antioxidant activity. Polymeric polyphenols showed higher antioxidant capacity compared with simple monomeric phenols. Furthermore, the number and position of hydroxyl groups in the benzene ring affected the antioxidant potential^[^^42^^]^. Other studies have evaluated the effect of various solvents on the RP of plant extracts. For example, Kchaou *et al.*^[^^39^^]^ showed that the best RP was observed for *Phoenix dactylifera* L. when the acetone/H_2_O solvent used. Furthermore, Metrouh-Amir *et al.*^[^^43^^]^ indicated that the most significant RP of *Matricaria pubescens *was found for the aqueous acetone extract.

Diabetes, as a chronic disease, is characterized by high blood glucose levels, particularly after fasting and consumption of meals. The inhibition of α-amylase as an enzyme involved in the hydrolysis of starch and oligosaccharides, has been considered as an important strategy for the management of diabetes. As illustrated in Table 4, the AAI rate of the acetone extract was significantly higher than the other extracts (*p* < 0.05). Since the acetone extract possessed the highest level of phenolic contents (Table 1), it appears that the presence of phenolic compounds might contribute to AAI. Different studies have revealed that phenolics-rich extracts exhibited a good inhibitory effect on α-amylase^[^^44^^,^^45^^]^. 

Inhibition of AChE, the critical enzyme in the *hydrolysis of acetylcholine*, is the first-line therapy for Alzheimer’s diseases^[^^46^^]^. Among the tested extracts, the acetone showed the highest AChE inhibitory activity, which were consistent with the findings of Sarikurkcu *et al.*^[^^1^^]^ regarding the inhibitory capability of the acetone extract of *Clinopodium vulgare*. To summarize, acetone extract exhibited the highest inhibition effect against both key enzymes when compared with two other extracts, probably due to its highest level of phenolics (Table 1).

**Table 6:** Correlation coefficients between the data obtained from different assays

**Evaluation** **method**	**Total bioactive component**				**Antioxidant activity**					**Enzyme inhibitory activity**	
	**TPC**	**TFC**	**TAC**		**DPPH**	**FRAP**	**IT**_50_	**EC**_50_		**AAI**	**AChEI**
TPC	1	-	-		-	-	-	-		-	-
TFC	0.767^*^	1	-		-	-	-	-		-	-
TAC	-0.799^**^	-0.679^*^	1		-	-	-	-		-	-
DPPH	-0.841^**^	-0.917^**^	0.662^ns^		1	-	-	-		-	-
FRAP	0.802^**^	0.932^**^	-0.663^ns^		-0.918^**^	1	-	-		-	-
IT_50_	-0.831^**^	-0.928^**^	0.691^*^		0.951^**^	-0.972^**^	1	-		-	-
EC_50_	-0.750^*^	-0.926^**^	0.533^ns^		0.944^**^	-0.944^**^	0.959^**^	1		-	-
AAI	-0.915^**^	-0.930^**^	0.810^**^		0.937^**^	-0.916^**^	0.939^**^	0.905^**^		1	-
AChEI	-0.787^*^	-0.942^**^	0.555^ns^		0.953^**^	-0.961^**^	0.969^**^	0.982^**^		0.904^**^	1

^*^Correlation is significant at the 0.05 level (2-tailed); ^**^Correlation is significant at the 0.01 level (2-tailed); ns, not significant

As the herbal remedies are generally safe with fewer side effects, pharmacologically active constituents from traditional *medicinal* *plants* have received much attention of pharmaceutical and scientific communities. The antimicrobial activity of the various extracts of *A. repens* against different strains of bacteria supported the scientific validity of the plant being used traditionally as a medicine. Like other studies, herein, the inhibition zones enhanced with the increasing content of polyphenolic compounds^[^^47^^]^. 

As critical bioactive constituents of plants, phenolic compounds, flavonoids, tannins, and alkaloids play key roles in the antibacterial properties of plants. Some of these bioactive compounds inhibit the life processes of microorganisms by altering their biochemical systems, binding their protein molecules, acting as chelating agents, or causing inflammation of the cells^[^^48^^]^. Phenolic compounds could present their antibacterial ability via interaction with enzymes, abrogation synthesis of nucleic acids, deprivation of metal ions, adsorption to the cell membrane, and disruption of the permeability barrier of bacterial cell envelopes^[^^49^^]^. Humeera *et al.*^[^^50^^]^ have suggested that the high content of phenolic compounds in the methanolic extract of *Rumex dentatus* leads to its high antibacterial activity. The Gram-negative bacteria studied herein were not sensitive to the plant extracts. These variations might be associated with the differences between the cell surface structures of Gram-negative and Gram-positive bacteria^[^^51^^]^.

The correlation coefficients between the antioxidant, enzyme inhibitory activities, and the TPC, flavonoid content, and TAC for all extracts were analyzed (Table 6). According to the results, a strong correlation was found between phenolic compounds and the antioxidant activities assessed by various methods, evidencing that phenolic compounds are the major constituents that contribute to the antioxidant activity. The antioxidant capacity determined by the DPPH assay (IC_50_) showed a strong negative correlation with TPC (r = -0.841, *p* < 0.01) and TFC (r = -0.907, *p* < 0.01), confirming that the lower the amount of IC_50_, the higher would be the content of TFC and TPC. *A strong correlation* *was established* *between* phenolic compounds and ferric reducing/antioxidant power of all extracts (r = 0.802, *p* < 0.01 for TPC and r = 0.932, *p* < 0.01 for TFC). Furthermore, a strong negative relationship was found between TPC/TFC with the α-amylase and AChE inhibitory activity. The relationship between antioxidant activity and TPC has previously been reported^[^^52^^,^^53^^]^. The results obtained in the present study suggest that the phenolic compounds are the main components affecting the antioxidant activity, and the inhibitory enzyme activity of *A.* *repens* leaf extracts. Owing to the redox potential, phenolic compounds could act as key reductants, free radical scavengers, and singlet and triplet oxygen quenchers^[^^54^^]^. 

Medicinal herbs offer potent natural alternatives for synthetic antioxidants and disease-associated enzyme inhibitors. To our knowledge, this study was the first to evaluate the bioactivities of *A. repens* extracts prepared by different extraction solvents. Our data revealed that the extraction with different solvents affects the contents of phenolic compounds and their bioactivities. Among the extracts, the aqueous acetonic extract exhibited the highest phenolic content, antioxidant ability, anti-cholinesterase, and anti-amylase activity. Meanwhile, it would be interesting to identify the key compounds contributing to these activities to accurately evaluate the influence of different solvents on the extraction of phenolic constituents and the association between their compositions and the resulting activities. Taken together, *A. repens* extracts have been found to be a promising antioxidant-rich therapeutic component with antimicrobial and enzyme inhibitory features for the food and pharmaceutical industry. However, further studies are required to evaluate the safety and efficacy of *A. repens.*

## CONFLICT OF INTEREST.

None declared.
